# Impact of immune-related adverse events on response to neoadjuvant chemoimmunotherapy in triple-negative breast cancer: a single-institution retrospective study

**DOI:** 10.1007/s10549-026-07930-8

**Published:** 2026-02-23

**Authors:** Michelle Sterpi, Nechama Dreyfus, Yungtai Lo, Susan Fineberg, Harjot Gill, Della Makower

**Affiliations:** 1 Montefiore Einstein Comprehensive Cancer Center, Bronx, NY USA; 2 Albert Einstein College of Medicine, Bronx, NY USA

**Keywords:** Triple-negative breast cancer, Immune-related adverse events, Neoadjuvant chemotherapy, Tumor-infiltrating lymphocytes, Pembrolizumab

## Abstract

**Purpose:**

Immune-related adverse events (irAEs) have emerged as a potential surrogate marker for immunotherapy response across tumor types. We evaluated the association between irAEs and pathologic complete response (pCR) in a racially diverse cohort of patients with triple-negative breast cancer (TNBC) treated with neoadjuvant chemoimmunotherapy.

**Methods:**

We conducted a retrospective analysis of 46 patients with early-stage TNBC treated with neoadjuvant chemoimmunotherapy between January 2021 and March 2023 at a single NCI-designated Comprehensive Cancer Center. irAEs, tumor-infiltrating lymphocytes (TILs), and clinicopathologic characteristics were abstracted from the medical record. Associations with pCR were analyzed using Fisher’s exact and Wilcoxon rank-sum tests.

**Results:**

Among 46 patients, the median age was 60.5 years. Most identified as Black (n = 27, 58.7%) or Hispanic (n = 14, 30.4%). irAEs occurred in 13 patients (28.2%), most commonly hypothyroidism, rash, and arthritis. The pCR rate was 55.8% (24/43 evaluable patients). Patients who developed irAEs were more likely to achieve pCR (84.6% vs. 45.2%, p = 0.039). Higher TILs (median 29%) were associated with pCR both as a continuous variable (p = 0.004) and categorically (p = 0.002), but not with irAE development (p = 0.341). pCR was more common among Hispanic patients (p = 0.005), and inversely associated with Black race (p = 0.003) and older age (p = 0.028).

**Conclusion:**

IrAEs may serve as a surrogate for treatment response to neoadjuvant chemoimmunotherapy in early TNBC. Additionally, racial and age-based differences in treatment response suggest underlying immunologic or biologic variation. These findings highlight the importance of diverse cohort representation in immunotherapy studies and warrant validation in prospective trials.

Triple-negative breast cancer (TNBC) accounts for approximately 15–20% of all breast cancers and is defined by the absence of all three targetable breast cancer receptors: estrogen, progesterone and human epidermal growth factor receptor 2 (HER2) [1]. TNBC is an aggressive breast cancer subtype, with higher rates of early recurrence and poorer survival compared to other breast cancer types [2]. Black women are disproportionately affected by TNBC and continue to experience disparities in both incidence and outcomes despite treatment advances [2, 3].

The addition of immune checkpoint inhibitors (ICIs) to chemotherapy regimens has improved outcomes in early-stage TNBC. Pembrolizumab is a monoclonal antibody that inhibits programed cell death protein 1 (PD-1) on T-cells from binding with programmed death ligand 1 (PD-L1) which is expressed inappropriately on cancer cells as a mechanism of immune evasion. Binding of PD-1 to PD-L1 inactivates T-cells, therefore by inhibiting this interaction, the immune system remains active to recognize and target cancer cells. The KEYNOTE-522 trial demonstrated that adding pembrolizumab to neoadjuvant chemotherapy significantly improved pathologic complete response (pCR) rates and event-free survival, leading to its U.S. Food and Drug Administration (FDA) approval for stages II/III TNBC in 2021 [4, 5]. However, ICIs may lead to immune-related adverse events (irAEs), which range from mild to life-threatening and may result in permanent toxicity [6] 

Across tumor types, the development of irAEs has been associated with favorable outcomes, possibly reflecting enhanced immune activation [7]. In TNBC, however, small retrospective studies evaluating the relationship between irAEs and pCR have yielded conflicting results [8, 9]. Importantly, minority populations remain underrepresented in immunotherapy clinical trials, limiting the generalizability of findings. One large retrospective analysis of patients treated with ICIs for metastatic cancer reported both a lower incidence of irAEs and poorer survival in Black patients compared with white patients [10].

To address these gaps, we conducted a retrospective study of patients with early-stage TNBC treated with neoadjuvant chemoimmunotherapy (NCIT) at our institution, a National Cancer Institute–designated Comprehensive Cancer Center located in the Bronx, NY. Our patient population reflects the racial and ethnic diversity of the Bronx, a historically underserved borough where approximately 56% of residents identify as Hispanic or Latino and 29% as Black or African American [11]. This context offers an opportunity to evaluate patterns of treatment response and toxicity in populations that have been historically underrepresented in immunotherapy research.

We evaluated the frequency and severity of irAEs, their association with pCR, and potential demographic and clinicopathologic predictors of each. In particular, we examined the role of stromal tumor-infiltrating lymphocytes (TILs), which have been associated with both improved response to neoadjuvant therapy and higher pCR rates in TNBC [12], but whose relationship to irAEs remains unclear.

## Methods

### Study population and data collection

Patients who received NCIT for TNBC at our institution between 2021 and 2023 were identified from the pharmacy database. All patients received NCIT consisting of pembrolizumab combined with a taxane-based chemotherapy backbone. Regimens were categorized as KEYNOTE-522–like (weekly paclitaxel ± carboplatin followed by doxorubicin and cyclophosphamide with pembrolizumab). No patients received a NeoPACT-style docetaxel–carboplatin regimen. Demographic and clinical data, including body mass index (BMI), germline genetic data, tumor stage, histologic grade, HER2 immunohistochemistry (IHC) status, treatment regimen and treatment completion rates, were obtained by chart review. Germline genetic testing was categorized as negative, declined testing, pathogenic variant, or variant of uncertain significance (VUS). Mutations in BRCA1, BRCA2, and RAD51C were defined as pathogenic variants. Information on irAEs, including type and grade, was also obtained by chart review. IrAEs were graded according to the Common Terminology Criteria for Adverse Events (CTCAE), version 5.0.

### Evaluation of TILs

Stromal TILs were visually estimated within the borders of the invasive carcinoma on pretreatment biopsies, using the International TILs Working Group criteria. TILs were evaluated as a continuous variable, and were also categorized as ≤ 30%, 31–49%, or ≥ 50%.

### Definitions and variables

pCR was defined as no residual invasive disease in the breast or axilla (ypT0/Tis ypN0) following surgery. Completion of NCIT was categorized as: full course, > 80% of planned treatment, or did not complete treatment (< 80% of planned treatment). Reasons for early discontinuation of treatment were recorded as chemotherapy toxicity, irAE, progression of disease, or unknown.

### Statistical analysis

Associations of patient characteristics with pCR or irAE development were assessed using Wilcoxon rank-sum tests for age and TILs and chi-square or Fisher’s exact tests for race, ethnicity, T stage, N stage, grade, HER2 IHC status, TILs categorized as ≤ 30, 31–49, or ≥ 50%. and NCIT completion. An exploratory multivariable logistic regression model was performed to evaluate factors potentially associated with pCR. Given the modest sample size, the model was restricted to age, ethnicity, and the presence of an irAE, variables selected a priori based on clinical relevance. Results are interpreted as hypothesis-generating. All analyses were performed using SAS software, version 9.4 (SAS Institute, Cary, NC). A p < 0.05 was considered statistically significant.

## Results

### Patient population

Patient and tumor characteristics are summarized in Table 1. 46 patients met inclusion criteria and were included in this analysis. Median age at diagnosis was 60.5 (range, 33–88) years. 27 patients (58.7%) identified as Black, 3 (6.5%) as white, and 16 (34.8%) as other or declined to specify. Fourteen patients (30.4%) were Hispanic, 30 (65.2%) were non-Hispanic, and 2 (4.3%) identified as other or declined to specify. BMI was available for all 46 patients. Mean BMI was 31.1, median was 29.5, and range was 18–47. No significant association was observed between BMI and pCR or irAE development. Germline genetic status was available for all patients: 28 (60.9%) tested negative, 10 (21.7%) declined testing, 5 (10.9%) had a VUS, and 3 (6.5%) carried pathogenic variants (BRCA1, BRCA2, RAD51C).

**Table 1:** Patient Demographics, Tumor Characteristics, and Treatment Details

Variable	N (%) or median
Total patients	46
Age, median (range)	60.5 (33–88)
Race, n (%)	
Black	27 (58.7)
White	3 (6.5)
Other/declined	16 (34.8)
Ethnicity, n (%)	
Hispanic	14 (30.4)
Non-hispanic	30 (65.2)
Other/Declined	2 (4.3)
BMI, median (range)	29.5 (18–47)
Pathogenic genetic variant, n (%)	3 (6.5%)
Clinical tumor (T) stage, n (%)	
T1	2 (4.3)
T2	27 (58.7)
T3	12 (26.1)
T4	4 (8.7)
Local recurrence	1 (2.2)
Clinical node (N) stage, n (%)	
N0	29 (63.0)
N1	12 (26.1)
N2	2 (4.3)
N3	2 (4.3)
Anatomic stage (AJCC 8th), n (%)	
Stage IIA	20 (43.5)
Stage IIB	15 (32.6)
Stage IIIA	4 (8.7)
Stage IIIB	4 (8.7)
Stage IIIC	1 (2.2)
Not assigned	1 (2.2)
Histologic grade, n (%)	
Grade 3	42 (91.3)
Grade 2	3 (6.5)
HER2 IHC status, n (%)	
HER2 Negative	15 (32.6)
HER2 Low	31 (67.4)
IHC 1 +	16 (34.8)
IHC 2 +	15 (32.6)
TILs evaluable, n (%)	
Yes	30 (65.2)
No	16 (34.8)
Median TILs (%)	29
NCIT regimen, n (%)	
KEYNOTE-522–like regimen	46 (100)
NCIT completion, n (%)	
Full course	24 (52.2)
≥ 80% completed	7 (15.2)
< 80% completed	15 (32.6)
Achieved pCR, n (%)	24 (52.2)
irAE, n (%)	13 (28.2)
> 1 irAE	4 (8.7)
Adjuvant pembrolizumab, n (%)	31 (67.4)

*BMI* body mass index*, AJCC* American Joint Committee on Cancer, *HER2* human epidermal growth factor receptor 2, *IHC* immunohistochemistry, *TILs* tumor-infiltrating lymphocytes, *NCIT* neoadjuvant chemoimmunotherapy, *pCR* pathologic complete response, *irAE* immune-related adverse event.

Most patients presented with T2 tumors (n = 27, 58.7%), followed by T3 (n = 12, 26.1%) and T4 (n = 4, 8.7%). Two patients (4.3%) had T1 tumors, and one (2.2%) presented with local recurrence. Nodal staging showed 29 (63.0%) patients were clinically node-negative (N0), while 12 (26.1%) were N1, 2 (4.3%) were N2, and 2 (4.3%) were N3.

Using AJCC 8th Edition anatomic staging criteria, the most common clinical stage was Stage IIA (n = 20, 43.5%), followed by Stage IIB (n = 15, 32.6%). Four (8.7%) patients were staged as Stage IIIA, 4 (8.7%) as Stage IIIB, and 1 (2.2%) as Stage IIIC. One (2.2%) patient could not be assigned a clinical stage due to presentation with local recurrence, which fell outside standard AJCC staging definitions.

Based on HER2 IHC, 15 patients (32.6%) were HER2-negative, and 31 (67.4%) were HER2-low. Among the HER2-low group, 16 (34.8%) were IHC 1 + and 15 (32.6%) were IHC 2 + . Of the 43 patients included in the response analysis, 14 (32.6%) were HER2-negative and 29 (67.4%) were HER2-low (15 [34.9%] IHC 1 + , 14 [32.6%] IHC 2 +).

### Evaluation of TILs

Tumor-infiltrating lymphocytes (TILs) were evaluable in 30 patients (65.2%), including 28 (65.1%) in the final analysis. The median TILs percentage across the evaluable group was 29%. In two patients with multifocal tumors, the TILs percentage was calculated as the average of the individual tumor foci. When assessed by race, median TILs percentage was 27% (n = 18) for African American patients, 38.75% (n = 2) for white patients and 33% (n = 8) for those who registered race as other or declined.

### Chemoimmunotherapy regimens

Details of treatment regimens are summarized in Table 1. All patients received NCIT including pembrolizumab and a taxane, with 43 (93.5%) patients receiving paclitaxel and 3 (6.5%) receiving docetaxel. Thirty-five patients (76.1%) received doxorubicin, and 25 (54.3%) received carboplatin.

Thirty-one (67.4%) patients completed at least 80% of their planned NCIT regimen, including 24 (52.2%) who completed the full course and 7 (15.2%) who completed most of the treatment. Fifteen (32.6%) patients did not complete NCIT. Reasons for early discontinuation included chemotherapy-related toxicity (n = 9), irAE (n = 4), and progression of disease (n = 2). One patient had no clearly documented reason for discontinuation. Twenty (43.5%) patients received adjuvant pembrolizumab following surgery. Reasons for not receiving adjuvant therapy included clinical decline or death (n = 4), disease progression (n = 2), development of immune-related adverse events (n = 5), patient preference (n = 3), and loss to follow-up (n = 1).

### Immune-related adverse events

Details of irAE experienced by patients are summarized in Table 2. Thirteen (28.2%) patients developed at least one irAE, for a total of 18 individual events. Most patients experienced a single irAE, though several had multiple toxicities, and one patient developed a fatal event. The most frequently observed irAEs were dermatitis (n = 4) and hypothyroidism (n = 3). Other irAEs included adrenal insufficiency (n = 2), arthritis (n = 2), and hepatitis, panniculitis, myositis, transaminitis, pericarditis, pneumonitis, and encephalitis (n = 1 for each). Most patients experienced a single irAE, though 3 patients had 2 co-occurring toxicities: (hypothyroidism + dermatitis, arthritis + dermatitis, and pneumonitis + encephalitis) and one had 3 concurrent irAE (myositis, transaminitis, and pericarditis). Hepatitis was defined as clinical and radiologic hepatic inflammation whereas transaminitis referred to isolated enzyme elevation without clinical features.

**Table 2:** Type and Severity of Immune-Related Adverse Events (irAE) among all patients (N = 46)

IrAE	All Events^a^ (n = 18^b^)	All Events^a^ Grade ≥ 3 (n = 9)
Hypothyroidism	3	0
Rash	4	1
Adrenal Insufficiency	2	1
Arthritis	2	1
Hepatitis^c^	1	1
Panniculitis	1	0
Myositis	1	1
Transaminitis^c^	1	1
Pericarditis	1	1
Pneumonitis	1	1
Encephalitis	1	1

ᵃ Numbers in the table represent events, not patients.

ᵇ Thirteen patients (28.2%) developed at least one irAE, for a total of 18 individual events.

^c^ Hepatitis is defined as clinical and radiologic hepatic inflammation. Transaminitis is defined as isolated liver enzyme elevation without clinical features.

**Fig. 1:**
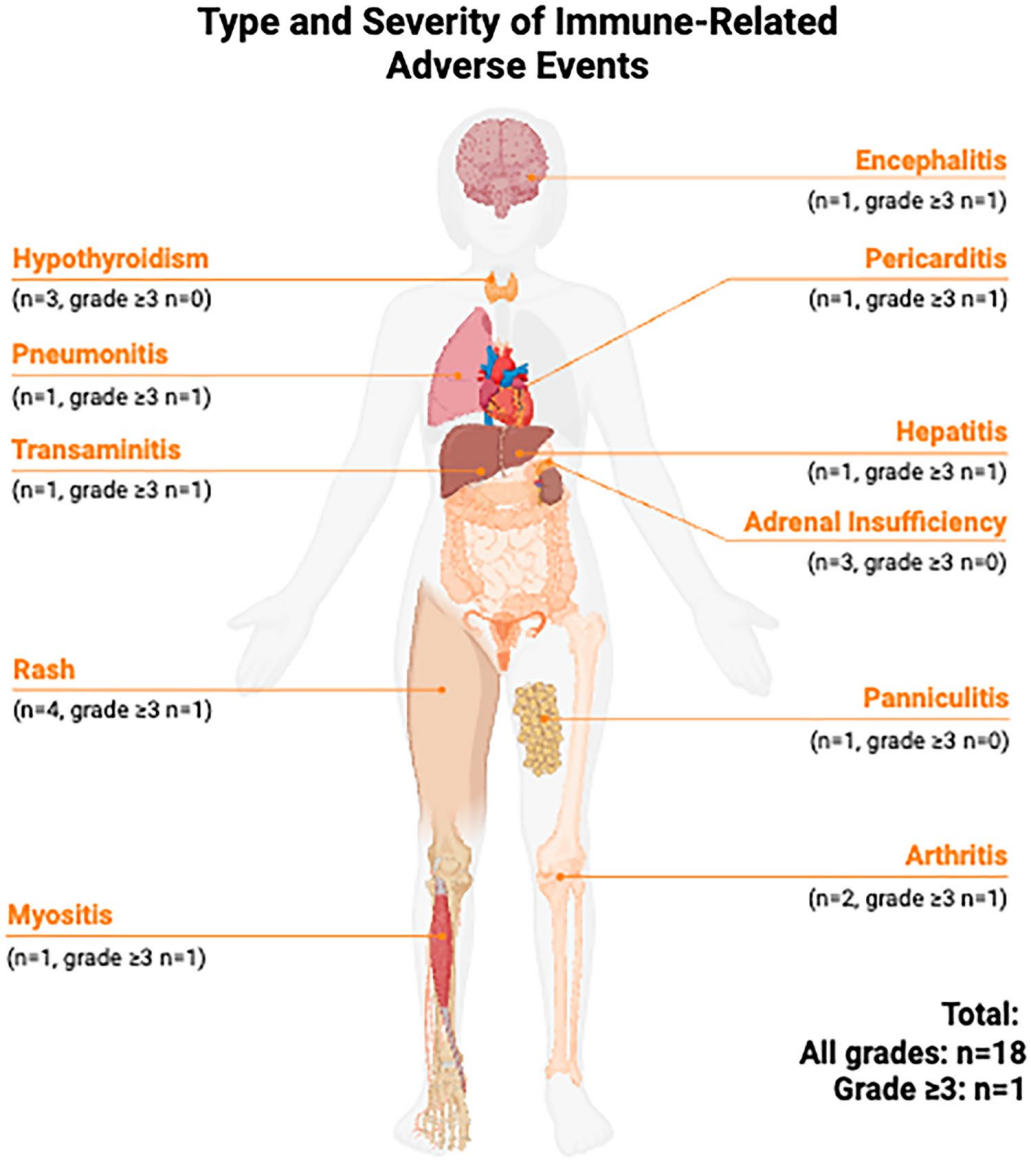
Anatomic distribution of immune-related adverse events (irAEs) in the study cohort (N = 46). Numbers indicate patients experiencing at least one event in each organ system

Five patients experienced only grade 1–2 irAEs, four experienced grade 3 toxicity, and one patient experienced simultaneous grade 3 and 4 events. One patient developed grade 5 irAE, consisting of pneumonitis and encephalitis. Two patients developed irAEs while receiving adjuvant pembrolizumab, following completion of NCIT and surgery. To aid visualization, Fig. 1 depicts the anatomic distribution of irAEs across affected organ systems.

### Response to treatment

Forty-one patients underwent surgery following NCIT. Surgical procedures included lumpectomy in 27 patients, mastectomy in 10, and bilateral mastectomy in 3. One patient underwent axillary lymph node dissection (ALND) only, having received NCIT for a locoregional recurrence. Reasons for not undergoing surgery included progression of disease (n = 2), fatal irAE (n = 1), patient refusal (n = 1), and unknown (n = 1). In total, 43 patients were evaluable for pCR, including two who experienced progression of disease on NCIT, and were assessed radiographically. Axillary surgery was performed in 42 patients (91.3%). One patient in our cohort who was treated with NCIT for locally recurrent breast cancer underwent breast surgery without axillary evaluation. 24 patients (52.2% of entire cohort; 55.8% of evaluable patients) achieved pCR.

### Factors associated with pCR and with development of irAE

Demographic and clinicopathologic features associated with pCR are summarized in Table 3. Among the 43 patients evaluable for response, development of irAE was significantly associated with achievement of pCR (p = 0.039). Other factors associated with pCR included younger age (p = 0.028), Hispanic ethnicity (p = 0.005), and higher stromal TILs (p = 0.005). Black race was negatively associated with pCR (p = 0.003). Completion of planned NCIT regimen and BMI were not associated with pCR. Of the three cases with pathogenic genetic variants (BRCA1, BRCA2 or RAD51C), one patient achieved a pCR and two did not. Given the small numbers, no meaningful analysis could be performed to assess for an association between pathogenic genetic variants and pCR.

**Table 3:** Associations of clinicopathologic characteristics and immune-related adverse events (irAEs) with pathologic complete response (pCR)

	No pCR N = 19	With pCR N = 24	p-value
Age, median (IQR)	64 (57, 68)	57 (48, 65)	0.028
Race, n (%) White Black Other/declined	1 (5.3) 16 (84.2) 2 (10.5)	2 (8.3) 9 (37.5) 13 (54.2)	0.003
Ethnicity, n (%) Non-Hispanic Hispanic Other / Declined	17 (89.5) 2 (10.5) 0	11 (45.8) 11 (45.8) 2 (8.3)	0.005
Clinical T stage, n (%) T1 T2 T3 T4	0 10 (55.6) 6 (33.3) 2 (11.1)	2 (8.3) 16 (66.7) 4 (16.7) 2 (8.3)	0.508
Clinical N stage, n (%) N0 N1 N2 N3	12 (66.7) 5 (27.8) 0 1 (5.5)	16 (66.7) 6 (25) 2 (8.3) 0	0.633
Nottingham score, n (%) Poorly Moderately	17 (89.5) 2 (10.5)	23 (100) 0	0.199
Her2 IHC, n (%) Negative Low	8 (42.1) 11 (57.9)	6 (25.0) 18 (75.0)	0.329
TILs%, median (IQR)	17.5 (8, 30)	54 (25, 59)	0.004
TILs ≤ 30% TILs 31–49% TILs ≥ 50%	11 (78.6) 3 (21.4) 0	4 (28.6) 2 (14.3) 8 (57.1)	0.002
NCIT completion, n (%) Full course ≥ 80% completed < 80% completed	8 (42.1) 4 (21.1) 7 (36.8)	16 (66.7) 2 (8.3) 6 (25)	0.244
irAE, n (%) No Yes	17 (89.5) 2 (10.5)	14 (58.3) 10 (41.7)	0.039

*irAE* immune-related adverse event, *pCR* pathologic complete response, *IQR* interquartile range*, T* tumor, *N* node, *HER2* human epidermal growth factor receptor 2, *IHC* immunohistochemistry, *TILs* tumor-infiltrating lymphocytes, *NCIT* neoadjuvant chemoimmunotherapy.

Associations of demographic and clinicopathologic features with the development of irAE are summarized in Table 4. No statistically significant associations were observed between irAE development and age, race, ethnicity, clinical anatomic tumor or nodal stage, histologic grade, HER2 IHC status (HER2 IHC 0 vs. HER2-low), BMI or completion of planned neoadjuvant therapy. Percentage of stromal TILs in biopsy specimen was not associated with development of irAE.

**Table 4:** Associations of clinicopathologic characteristics with immune-related adverse events (irAEs)

	No irAE N = 33	With irAE N = 13	p-value
Age, median (IQR)	62 (57, 68)	55 (48.5, 67))	0.249
Race, n (%) White Black Other/declined	2 (6.5) 21 (67.7) 8 (25.8)	1 (8.3) 4 (33.3) 7 (58.3)	0.081
Ethnicity, n (%) Non-hispanic Hispanic Other/declined	22 (71.0) 8 (25.8) 1 (3.2)	6 (50) 5 (41.7) 1 (8.3)	0.264
Clinical T stage, n (%) T1 T2 T3 T4	1 (3.3) 18 (60) 8 (26.7) 3 (10)	1 (8.3) 8 (66.7) 2 (16.7) 1 (8.3)	0.875
Clinical N stage, n (%) N0 N1 N2 N3	20 (66.7) 7 (23.3) 2 (6.7) 1 (3.3)	8 (66.7) 4 (33.3) 0 0	0.897
Nottingham score, n (%) Poorly Moderately	30 (100) 0	10 (83.3) 2 (16.7)	0.077
Her2 IHC, n (%) Negative Low	14 (45.2) 17 (54.8)	4 (33.3) 8 (66.3)	0.732
Tils%, median (IQR)	27 (12.5, 50.3)	42.5 (24.5, 60)	0.185
TILs ≤ 30% TILs 31–49% TILs ≥ 50%	12 (60) 4 (20) 4 (20)	3 (37.5) 1 (12.5) 4 (50)	0.341
NCIT completion, n (%) Full course ≥ 80% completed < 80% completed	17 (54.8) 4 (12.9) 10 (32.3)	7 (58.3) 2 (16.7) 3 (25.0)	1.0
pCR, n (%) No Yes	17 (54.8) 14 (45.2)	2 (16.7) 10 (83.3)	0.039

*irAE* immune-related adverse event, *IQR* interquartile range, *T* tumor, *N* node, *HER2* human epidermal growth factor receptor 2, *IHC* immunohistochemistry, *TILs* tumor-infiltrating lymphocytes, *NCIT* neoadjuvant chemoimmunotherapy, *pCR* pathologic complete response*.*

ᵃOne patient experienced a fatal irAE and is included in the “With irAE” group.

### Multivariable analysis

In multivariable logistic regression analysis assessing factors associated with the development of a pCR, age was not significantly associated with the outcome (OR 0.95; 95% CI 0.89–1.02; *p* = 0.189). Hispanic ethnicity was independently associated with higher odds of developing a pCR compared with non-Hispanic individuals (OR 6.88; 95% CI 1.16–40.72; *p* = 0.034). Patients who experienced an immune-related adverse event (iRAE) also had higher odds of developing a pCR, although this association did not reach statistical significance (OR 5.15; 95% CI 0.80–33.13; *p* = 0.084) (Table 5).

**Table 5:** Logistic regression analysis of factors associated with pathological complete response (pCR)

	Odds ratio (95% C.I.)	P value
Age, per year	0.95 (0.89, 1.02)	0.189
Hispanic vs Non-Hispanic (reference)	6.88 (1.16, 40.72)	0.034
iRAE vs No iRAE (reference)	5.15 (0.80, 33.13)	0.084

*irAE* immune-related adverse event.

## Discussion

Addition of the PD-1 immune checkpoint inhibitor pembrolizumab to standard neoadjuvant chemotherapy is now standard of care for patients with early TNBC [13]. The KEYNOTE 522 trial, in which patients with TNBC with tumors larger than 2 cm or axillary nodal involvement were randomized to neoadjuvant chemotherapy plus pembrolizumab vs chemotherapy plus placebo, showed an improvement in pCR from 56 to 63% (P < 0.001), event free survival at 36 months from 76.8 to 84.5% (HR 0.63; P < 0.001) and overall survival at 60 months from 81.7 to 86.6% (P = 0.002) in the pembrolizumab arm [4, 14, 15]. Despite these improved outcomes, the impacts of irAEs remain concerning. IrAEs are known side effects of immunotherapy, may affect any organ, range in severity from mild to life-threatening, and can be permanent [16–18]. We therefore sought to evaluate demographic and clinicopathologic features associated with increased likelihood of development of irAE and with increased likelihood of achievement of pCR in patients with TNBC receiving NCIT.

Our study found that patients who developed irAE after NCIT were significantly more likely to achieve pCR (p = 0.039) with an OR of 5.15 (95% CI 0.80–33.13; *p* = 0.084). The only clinicopathologic factors more strongly associated with pCR in our patient cohort were higher stromal TILs (p = 0.004) and younger age (p = 0.030), though the OR was not found to be significant. While we also noted an association between self-reported Hispanic ethnicity and pCR (p = 0.005, OR 6.88; 95% CI 1.16–40.72; p = 0.034), and a negative association between self-reported Black race with pCR (p = 0.003), the small size of our cohort, coupled with the number of patients who declined to self-report race, may influence these findings. Tumor stage, grade, BMI, HER2 IHC status, and completion of planned NCIT regimen were not significantly associated with pCR in our cohort. In addition, we did not identify any demographic or clinicopathologic factors that predicted for development of irAE. In particular, percentage of stromal TILs were not associated with development of irAE.

Our findings have several similarities and differences when compared with previously published data, which may be due to the size of our patient cohort, the retrospective nature of our evaluation, or the inclusion of a more diverse patient population than is generally included in clinical trials. For example, in the KEYNOTE 522 trial, any grade irAE occurred in 33.5% of patients in the treatment arm compared to 11.3% in the placebo group [4]; however, real-world data has shown much higher rates of irAE of 64–73% [8, 19–21]. Our cohort demonstrated a rate of any-grade irAE of 28.2%, similar to that seen in KEYNOTE 522, but lower than real-world data. Of note, the racial composition of patients enrolled on KEYNOTE 522 was 64% white, 20% Asian, 4.5% Black, and 1.8% American Indian or Alaska Native [4]. Information on ethnicity was not collected [22]. Data evaluating racial associations with irAE are limited, with mixed findings. One previous study found higher incidence of irAE in white patients across any stage IV solid malignancy treated with ICIs [10], while another study evaluating patients with advanced NSCLC found no difference in rates of irAE between non-Hispanic Black patients and other racial subgroups [23].

Our finding that irAE is strongly associated with pCR is similar to data in other tumor histologies that demonstrate improved cancer outcomes after ICI in patients who develop irAE [7, 24]. The data specific to breast cancer, however, is more limited. Foldi et al. initially found no association between irAE and pCR among 67 TNBC patients receiving neoadjuvant durvalumab plus chemotherapy, in a Phase I/II clinical trial that purposefully extended accrual of Black patients to ultimately comprise 31% of the study population [9]. Further follow-up of the trial, however, did show that patients who developed irAE had improved event-free survival [25]. In a small retrospective evaluation of 35 TNBC patients receiving the KEYNOTE 522 regimen at 3 centers in Austria and England, a significant association between irAE and pCR was found, with pCR occurring in 72.2% of patients who developed irAE, but only 30.8% of patients who did not [8]. On the other hand, in a recent retrospective study of 415 patients treated with NICT, no difference was seen between irAE and pCR [26]. Notably a weakness of that study was the ability to distinguish between irAE and chemotherapy-associated adverse events, thereby limiting the reliability of this finding [26]. In this new landscape, our study adds to an emerging body of evidence demonstrating a link between irAE and improved outcomes in TNBC.

We found that higher levels of stromal TILs in pretreatment biopsy specimens were strongly associated with pCR to NCIT, similarly to multiple prior reports [27–30]. The impact of stromal TILs on response to neoadjuvant therapy and on breast cancer prognosis is well described, although some researchers have found that this association is somewhat attenuated in Black women [31, 32]. In contrast, we found no association between high levels of stromal TILs and development of irAE. Similar evaluations in other tumor types have yielded inconsistent results. One study evaluating features of tumor immune microenvironment in non-small cell lung cancer found a strong correlation between TIL level and irAE [33], while similar evaluations in melanoma found no such associations [34, 35].

We also found higher pCR rates in women of Hispanic ethnicity, and lower pCR rates in Black women. These findings must be interpreted with caution, as the small number of white patients in our cohort and the number of patients who declined to self-report race may influence the observed associations. Nonetheless, they are supported with findings from a large real-world analysis of over forty thousand women using NCDB data [36]. Of note, in the recent retrospective review of 415 patients specifically treated with NICT referenced earlier, there was no reported difference in pCR based on race [26]. The relationship between race and breast cancer outcomes is multifactorial, and includes disparities in access to care [37–39], decreased treatment adherence and tolerance [40–43], and differences in tumor microenvironment [44, 45].

In summary, our study found that TILs and irAE, as well as age, race, and ethnicity, were associated with achievement of pCR to NCIT, while tumor stage and grade, BMI and completion of planned NCIT, were not. We found no factors that predicted for development of irAE, including presence of TILs in biopsy specimen. Limitations of our study include its small size and retrospective nature. Given the small sample size, the logistic regression estimates were imprecise, resulting in wide confidence intervals and a borderline association between iRAEs and pCR (p = 0.084). Although this trend suggests a possible relationship, the evidence remains insufficient for a definitive conclusion. Larger, adequately powered studies are needed to confirm whether iRAEs are truly associated with pathological response. Strengths of our study include a diverse cohort of patients typically underrepresented in clinical trials. Our study adds to previously published work regarding associations between irAE, TILs, and response to chemo-immunotherapy.

## Conclusion

Development of irAEs and higher stromal TILs were both associated with achievement of pCR to neoadjuvant chemoimmunotherapy in this diverse TNBC cohort, while no clinicopathologic features predicted for irAE occurrence. These findings reinforce the potential link between immune activation, toxicity, and efficacy of chemoimmunotherapy, and underscore the importance of studying underrepresented populations in immunotherapy research.

## Data Availability

The datasets generated and/or analyzed during the current study are not publicly available due to institutional policies and patient privacy protections but are available from the corresponding author on reasonable request.
